# Supplementary material for: Assessment of Microcirculatory Dysfunction by Measuring Subcutaneous Tissue Oxygen Saturation Using Near-Infrared Spectroscopy in Patients with Circulatory Failure
Source: Diagnostics (Basel). 2024 Oct 30;14(21):2428. doi: 10.3390/diagnostics14212428 (PMC11545383; doi:10.3390/diagnostics14212428)
Supplement: Supplementary file 1 [file diagnostics-14-02428-s001.zip › diagnostics-3198472-supplementary.pdf]

Supplementary Materials

Figure S1. Post-ICU admission changes in each parameter in the circulatory failure group.

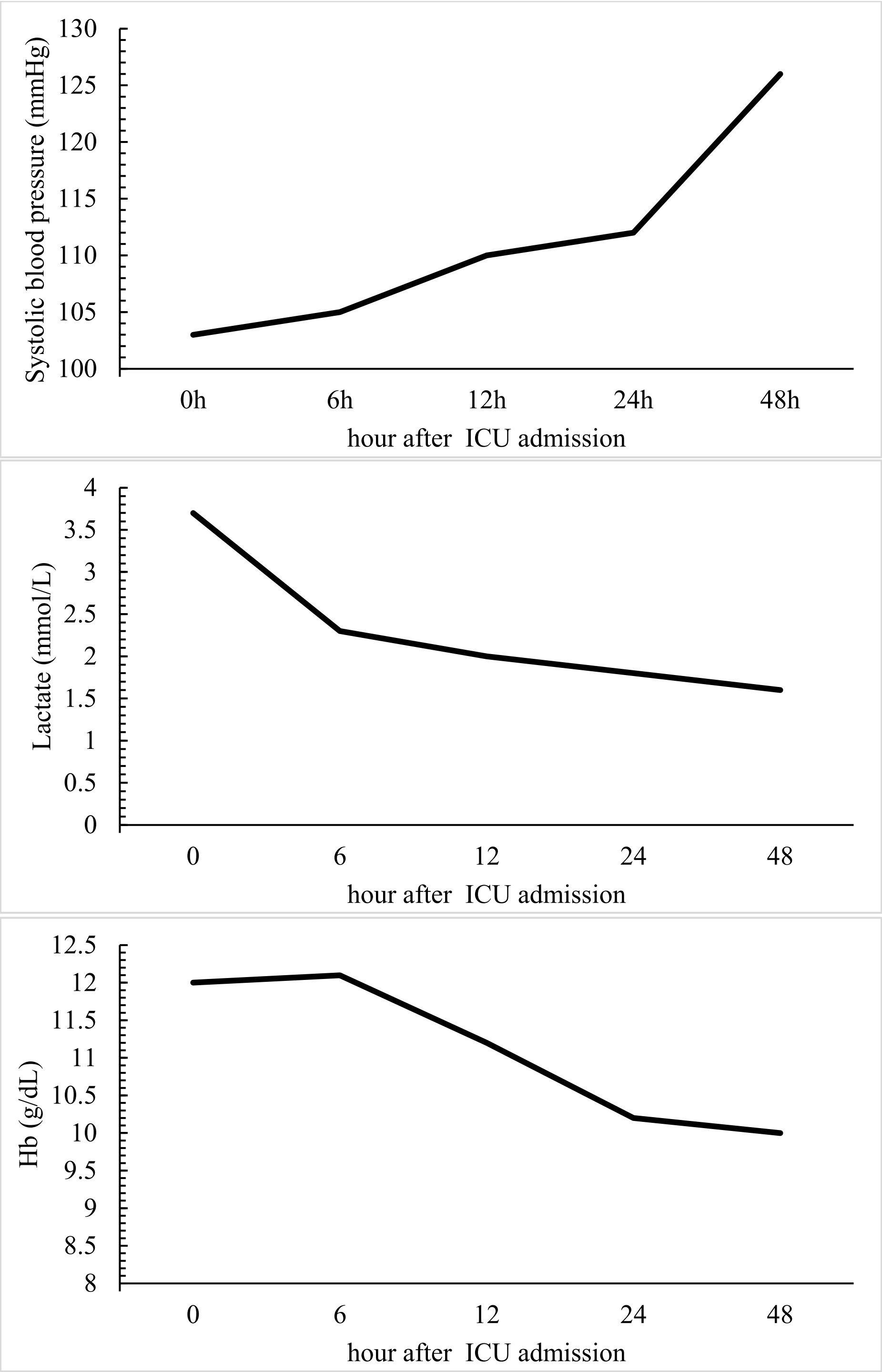

ICU = intensive care unit, Hb = hemoglobin,

After entering ICU, the systolic blood pressure tended to increase, and lactate and Hb levels tended to decrease.

Figure S2. Correlation of rSO_2 with systolic blood pressure, lactate, and hemoglobin in the circulatory failure group.

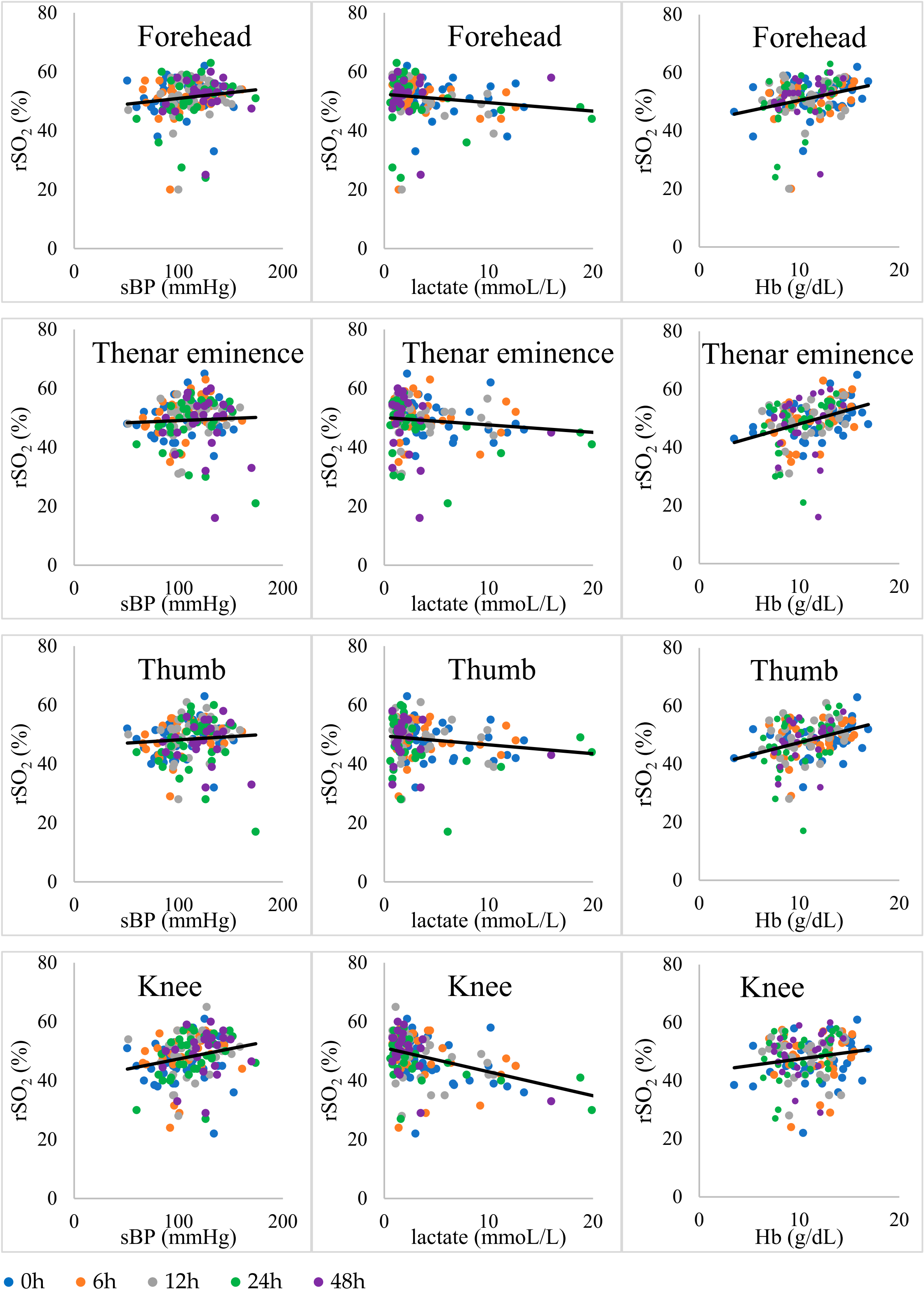

			Forehead	Thenar eminence	Thumb	Knee
Systolic blood pressure	0 hour	ϱ	0.12	0.12	0.26	0.14
	(n = 37)	p	0.46	0.33	0.12	0.41
	6 hour	ϱ	0.09	0.25	0.27	0.27
	(n = 35)	p	0.61	0.15	0.11	0.11
	12 hour	ϱ	0.18	0.18	0.17	0.21
	(n = 35)	p	0.31	0.30	0.33	0.22
	24 hour	ϱ	0.29	0.29	0.26	0.38
	(n = 33)	p	0.10	0.10	0.14	0.03*
	48 hour	ϱ	0.16	0.01	0.14	0.25
	(n = 24)	p	0.47	0.95	0.52	0.25
Lactate	0 hour	ϱ	-0.23	-0.25	-0.33	-0.43
	(n = 37)	p	0.18	0.13	0.05*	<0.01*
	6 hour	ϱ	-0.25	0.03	-0.19	-0.30
	(n = 34)	p	0.15	0.85	0.27	0.09
	12 hour	ϱ	-0.37	-0.12	-0.24	-0.40
	(n = 32)	p	0.04*	0.51	0.19	0.02*
	24 hour	ϱ	-0.22	-0.43	-0.28	-0.50
	(n = 33)	p	0.22	0.02*	0.11	<0.01*
	48 hour	ϱ	0.15	-0.13	0.08	-0.20
	(n = 22)	p	0.51	0.55	0.72	0.36
Hb	0 hour	ϱ	0.29	0.40	0.36	0.32
	(n = 37)	p	0.09	0.01*	0.03*	0.06
	6 hour	ϱ	0.33	0.37	0.24	0.14
	(n = 35)	p	0.06	0.03*	0.18	0.45
	12 hour	ϱ	0.88	0.27	0.28	0.12
	(n = 35)	p	0.63	0.14	0.12	0.52
	24 hour	ϱ	0.41	0.57	0.44	0.37
	(n = 33)	p	< 0.01	< 0.01*	0.01*	0.04*
	48 hour	ϱ	0.52	0.31	0.58	0.39
	(n = 24)	p	0.01*	0.14	< 0.01	0.07

rSO_2 = tissue saturation of oxygen; sBP = systolic blood pressure; Hb = hemoglobin

This table shows the correlation coefficients (ϱ) and *p*-values for rSO_2 at each site measured at 0, 6, 12, 24, and 48 h after admission and for systolic blood pressure, lactate, and Hb at the same

time. Correlation analysis was performed using the Spearman rank correlation coefficient.
* $p < 0.05$ indicates a significant correlation.

The rSO_2 had almost no significant correlation with blood pressure; however, significant correlations were often found between lactate and Hb levels.

Figure S3: Correlation between rSO2 and various organ damage indicators in the circulatory failure group.

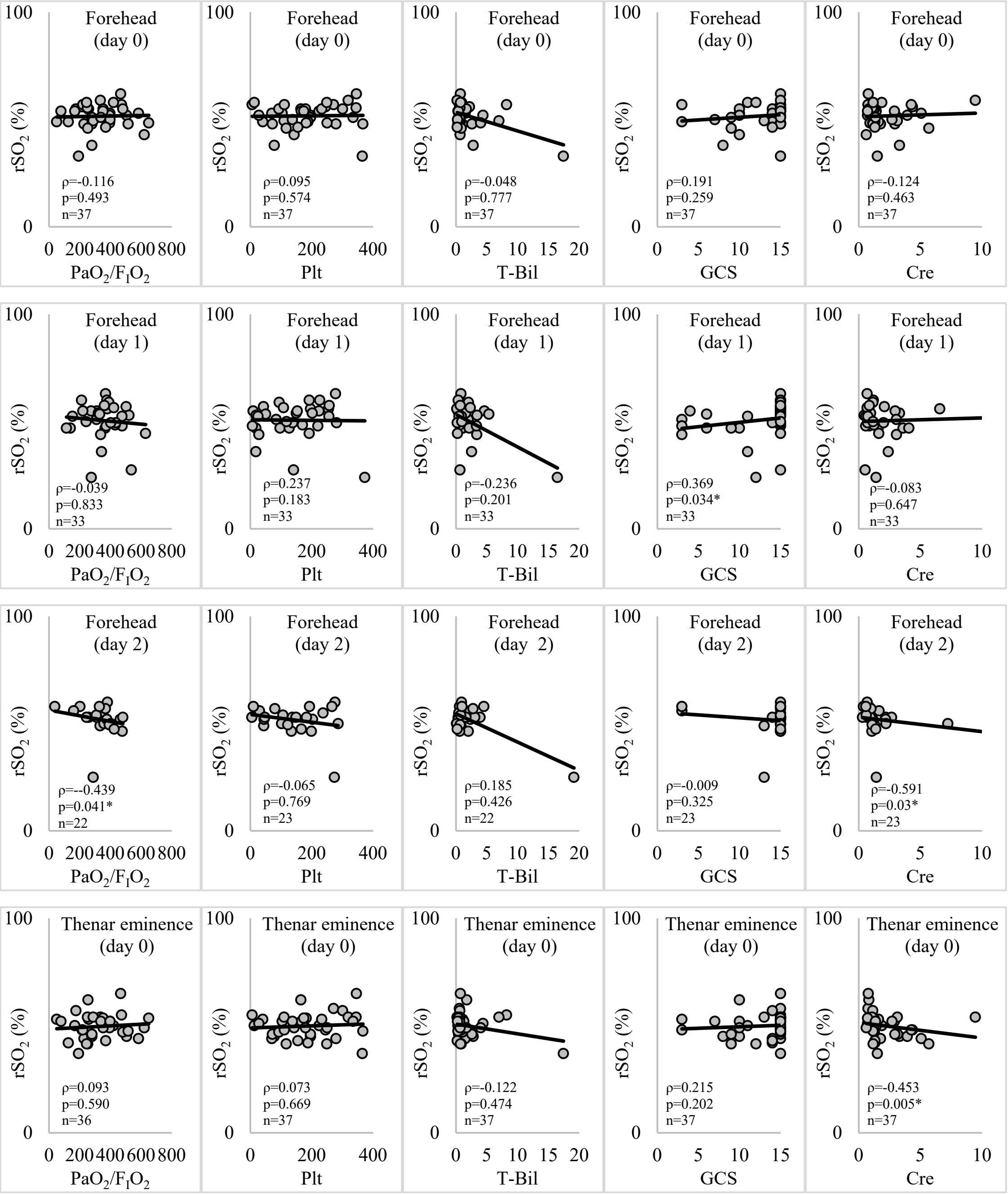

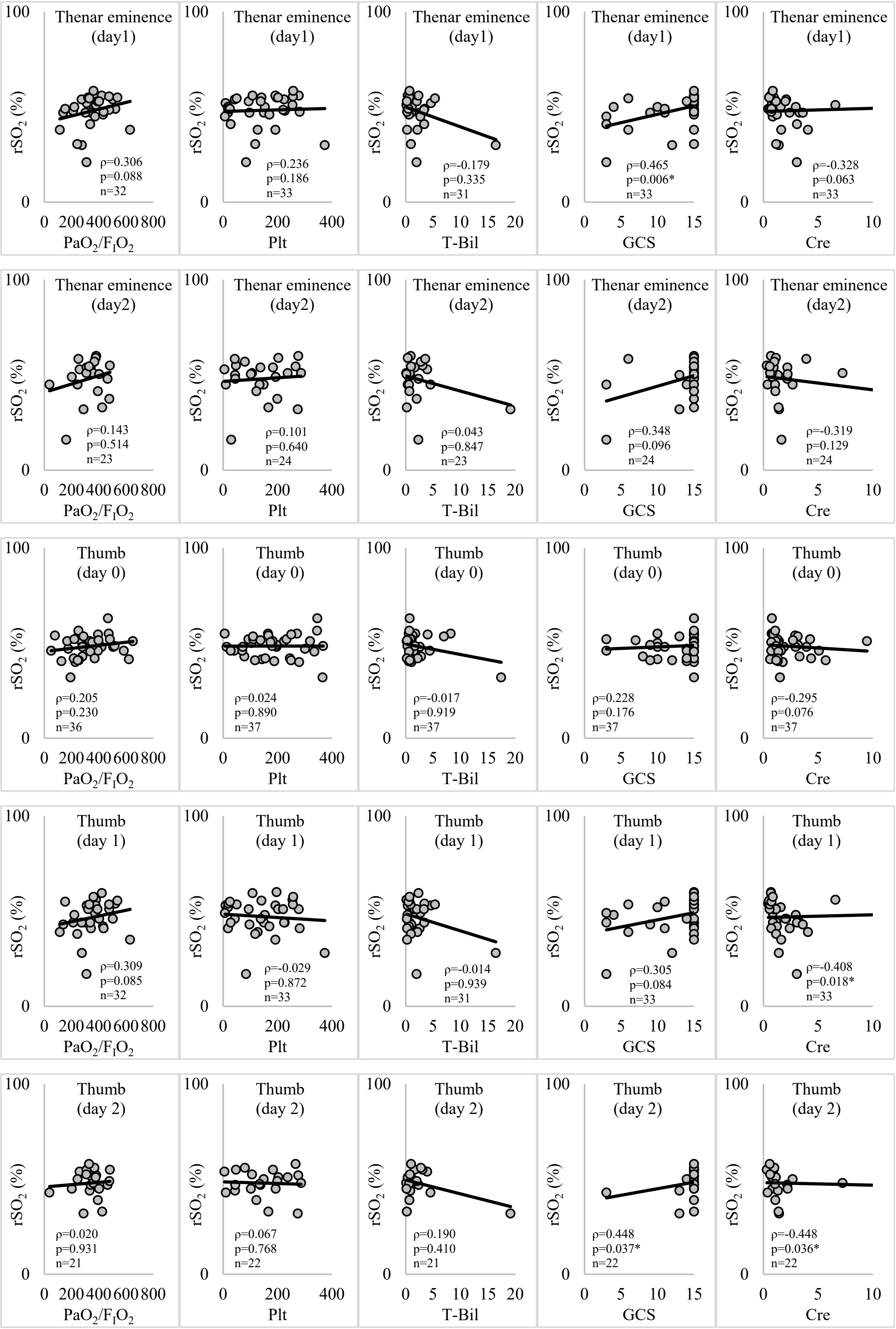

Thenar eminence (day1)
rSO2 (%)
100
0
ρ=0.306
p=0.088
n=32
0 200 400 600 800
PaO2/FIO2
Thenar eminence (day1)
ρ=0.236
p=0.186
n=33
0 200 400
Plt
Thenar eminence (day1)
ρ=-0.179
p=0.335
n=31
0 5 10 15 20
T-Bil
Thenar eminence (day1)
ρ=0.465
p=0.006*
n=33
0 5 10 15
GCS
Thenar eminence (day1)
ρ=-0.328
p=0.063
n=33
0 5 10
Cre
Thenar eminence (day2)
ρ=0.143
p=0.514
n=23
PaO2/FIO2
Thenar eminence (day2)
ρ=0.101
p=0.640
n=24
Plt
Thenar eminence (day2)
ρ=0.043
p=0.847
n=23
T-Bil
Thenar eminence (day2)
ρ=0.348
p=0.096
n=24
GCS
Thenar eminence (day2)
ρ=-0.319
p=0.129
n=24
Cre
Thumb (day 0)
ρ=0.205
p=0.230
n=36
PaO2/FIO2
Thumb (day 0)
ρ=0.024
p=0.890
n=37
Plt
Thumb (day 0)
ρ=-0.017
p=0.919
n=37
T-Bil
Thumb (day 0)
ρ=0.228
p=0.176
n=37
GCS
Thumb (day 0)
ρ=-0.295
p=0.076
n=37
Cre
Thumb (day 1)
ρ=0.309
p=0.085
n=32
PaO2/FIO2
Thumb (day 1)
ρ=-0.029
p=0.872
n=33
Plt
Thumb (day 1)
ρ=-0.014
p=0.939
n=31
T-Bil
Thumb (day 1)
ρ=0.305
p=0.084
n=33
GCS
Thumb (day 1)
ρ=-0.408
p=0.018*
n=33
Cre
Thumb (day 2)
ρ=0.020
p=0.931
n=21
PaO2/FIO2
Thumb (day 2)
ρ=0.067
p=0.768
n=22
Plt
Thumb (day 2)
ρ=0.190
p=0.410
n=21
T-Bil
Thumb (day 2)
ρ=0.448
p=0.037*
n=22
GCS
Thumb (day 2)
ρ=-0.448
p=0.036*
n=22
Cre

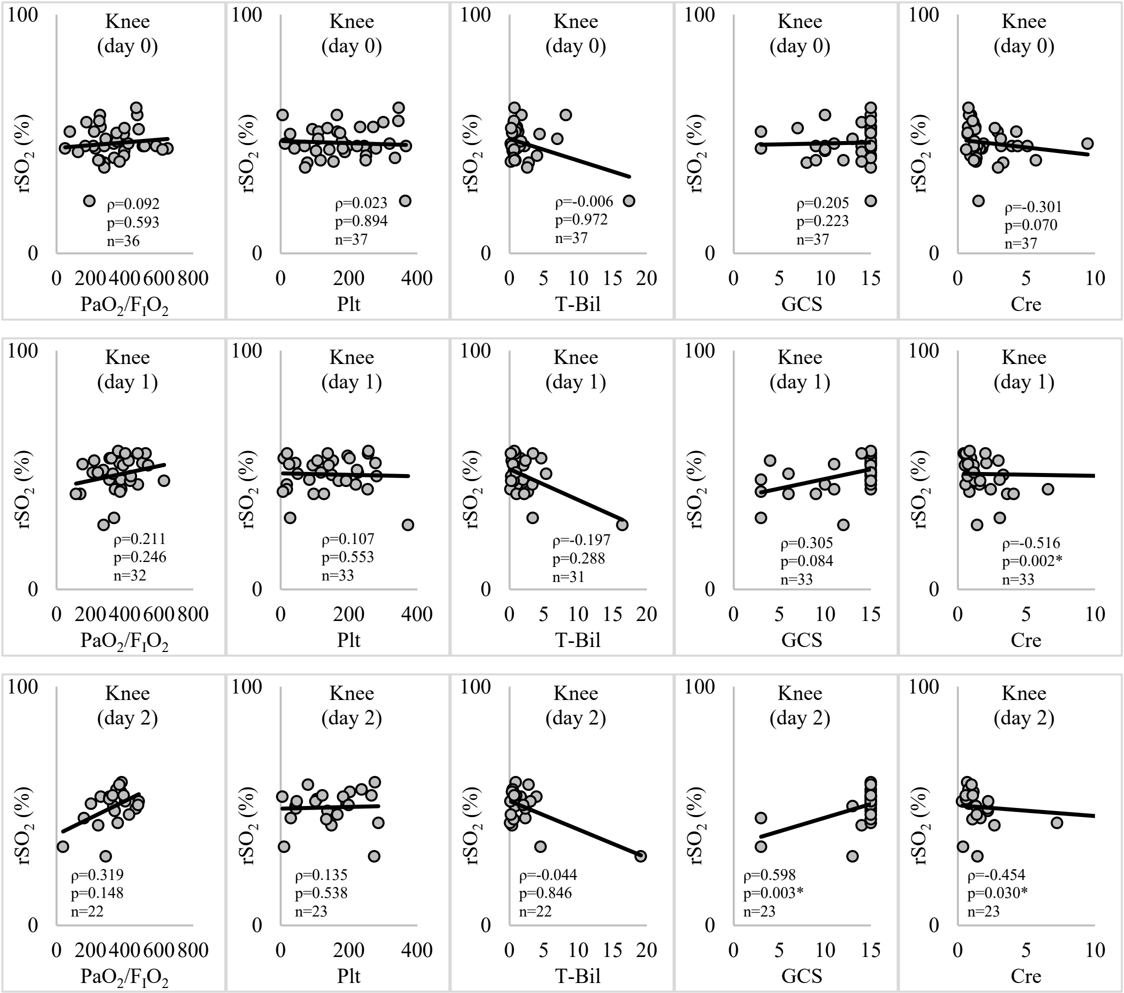

rSO_2 = tissue saturation of oxygen; PaO_2/F_IO_2 = partial pressure of arterial oxygen / fraction of inspired oxygen; Plt = platelet; T-Bil = total-bilirubin; GCS = Glasgow Coma Scale; Cre = creatinine

Correlation analysis was performed using the Spearman rank correlation coefficient. ϱ indicates the rank correlation coefficient. * $p < 0.05$ indicates a significant correlation.

This figure shows the correlation between rSO_2 at each site measured on Days 0, 1, and 2 after admission and various organ damage indicators (PaO_2/F_IO_2, Plt, T-Bil, GCS, and Cre). rSO_2 was frequently correlated with GCS and Cre.

Table S1. Multiple regression analysis on rSO_2 in the circulatory failure group.

Outcome variables	Independent variables	B	Standard error	β	t	*P*	95% confidence interval	VIF
rSO_2 at the forehead (48 h)	Age	-0.333	0.078	-0.859	-4.295	< 0.001*	-0.496–-0.171	1.572
	Hb (0 h)	-0.421	0.573	-0.126	-0.734	0.472	-1.620–0.778	1.163
	SOFA (0 h)	-0.915	0.280	-0.643	-3.262	0.004*	-1.502–-0.328	1.528
	R = 0.719, R^2 = 0.517, Adjusted R^2 = 0.440, F = 6.771, *P* = 0.003*							
rSO_2 at the thenar eminence (0 h)	Age	-0.063	0.048	-0.190	-1.301	0.202	-0.161–0.036	1.072
	Hb (24 h)	0.694	0.266	0.373	2.607	0.014*	0.152–1.235	1.026
	SOFA (24 h)	-0.464	0.207	-0.323	-2.237	0.032*	-0.886–-0.042	1.049
	R = 0.585, R^2 = 0.342, Adjusted R^2 = 0.282, F = 5.724, *P* = 0.003*							
rSO_2 at the thumb (0 h)	Age	-0.075	0.050	-0.220	-1.494	0.145	-0.176–0.027	1.072
	Hb (48 h)	0.601	0.274	0.316	2.191	0.036*	0.043–1.160	1.026
	SOFA (48 h)	-0.502	0.214	-0.342	-2.346	0.025*	-0.938–-0.067	1.046
	R = 0.576, R^2 = 0.332, Adjusted R^2 = 0.271, F = 5.459, *P* = 0.004*							
rSO_2 at the thumb (48 h)	Age	-0.186	0.088	-0.478	-2.127	0.048*	-0.370–-0.002	1.450
	Hb (48 h)	0.673	0.672	0.208	1.001	0.330	-0.740–2.085	1.235
	SOFA (48 h)	-0.744	0.368	-0.464	-2.022	0.058	-1.518–0.029	1.508
	R = 0.610, R^2 = 0.372, Adjusted R^2 = 0.268, F = 3.559, *P* = 0.035*							

rSO_2, regional tissue oxygen saturation; sBP, systolic blood pressure; Hb, hemoglobin; SOFA, sequential organ failure assessment; VIF, variance inflation factor.

Each independent factor was confirmed not to be multicollinear by VIF.

The regression equations for the forehead (0 and 24 h), thenar eminence (24 and 48 h), and thumb (24 h) are not shown in the table because significant equations could not be calculated for the following reasons: unstandardized residuals in the forehead (0 and 24 h), thenar eminence (24 h), and thumb (24 h) were confirmed to be non-normally distributed using the Shapiro–Wilk test. The p-values of the regression equations for the thenar eminence (48 h) and thumb (24 h) were > 0.05.

* $p < 0.05$, indicating a significant correlation.

The rSO_2 at the forehead (48 h), thenar eminence (0 h), and thumb (0 h) were significantly related to the SOFA score. The rSO_2 at the forehead (48 h) and thumb (48 h) were significantly associated with age. The rSO_2 at the thenar eminence (24 h) and thumb (0 h) were significantly related to Hb levels.
